# Profile of Cognitive Complaints in Vascular Mild Cognitive Impairment and Mild Cognitive Impairment

**DOI:** 10.1155/2013/865827

**Published:** 2013-10-28

**Authors:** Jenny Gu, Corinne E. Fischer, Gustavo Saposnik, Tom A. Schweizer

**Affiliations:** Keenan Research Centre of the Li Ka Shing Knowledge Institute of St. Michael's Hospital, 30 Bond Street, Toronto, ON, Canada M5B 1W8

## Abstract

*Objective*. Vascular mild cognitive impairment (VaMCI) is differentiated from mild cognitive impairment (MCI) by the presence of vascular events such as stroke or small vessel disease. Typically, MCI and VaMCI patients present with subjective complaints regarding cognition; however, little is known about the specific nature of these complaints. We aimed to create a profile of subjective cognitive complaints in MCI and VaMCI patients with similar levels of objective cognitive performance. *Methods.* Twenty MCI and twenty VaMCI patients were recruited from a Memory Disorders Clinic in Toronto. Subjective cognitive complaints were assessed and categorized using the Neuropsychological Impairment Scale. *Results.* MCI and VaMCI patients achieved similar scores on measures of objective cognitive function (*P* > 0.100). However, the VaMCI group had more subjective complaints than the MCI group (*P* = 0.050), particularly in the critical items, cognitive efficiency, memory, and verbal learning domains of the Neuropsychological Impairment Scale. *Conclusions*. Our findings support the idea that VaMCI and MCI differ in their clinical profiles, independent of neuroimaging. VaMCI patients have significantly more subjective cognitive complaints and may be exhibiting particular deficits in memory, verbal learning, and cognitive efficiency. Our findings promote the need for further research into VaMCI-specific cognitive deficits.

## 1. Introduction

 As adults age, it is common for cognitive problems to arise. Subjective cognitive complaints (SCC) are quite prevalent among older adults, with some estimates suggesting that between 25% and 50% of all older adults have self-perceived memory impairment [1, 2]. In clinical practice, it is often difficult to assess the veracity and severity of subjective cognitive complaints, primarily because such complaints vary widely from individual to individual. As a result, clinicians and caregivers perhaps do not consider subjective complaints to have the same weight as objective findings. However, studies have shown that subjective complaints may be valid indicators of current and future cognitive impairment. A recent study by Amariglio and colleagues showed that certain subjective complaints, such as “I have trouble finding my way around familiar streets,” are correlated with impairment in delayed recall, naming, and semantic fluency [3]. A review conducted by Jonker and colleagues showed that memory complaints may be predictive of dementia or Alzheimer's disease onset within two to four years, especially in individuals with a diagnosis of mild cognitive impairment (MCI) [1].

Subjective cognitive complaints also have clinical implications that are outside the realm of cognitive function. A review by Mol and colleagues [2] found that SCC correlated with depression, anxiety [4, 5], and low level of well-being, even in the absence of objective cognitive impairment. Fischer et al. [6] in a previous study compared SCC and objective cognitive function in older patients with and without major depression and found no changes in objective cognitive function between the two groups, but significantly more SCC in the depressed group. SCC have also been correlated with decreased functional ability, even when depression is controlled for [7]. Although the direction of causality between many of these variables remains unclear, SCC should be considered in a clinical setting because of their associations with objective impairment, dementia, depression, low quality of life, and functional ability. Previous research in the area of SCC has largely focused on memory complaints [1–3, 7, 8]. In the current study, we have broadened the scope of SCC in order to examine other areas of cognition that have shown impairment following complaints [3]. Previous studies have also used varying methods of evaluating SCC. The most common method appears to be a single, dichotomous, “yes or no” assessment of complaints (e.g., “Do you find that you have trouble with your memory?”) [7–13], or a combination of similar “yes or no” questions [3, 14, 15]. Furthermore, most of the existing literature on SCC examines community-dwelling, healthy, and older populations [3, 7, 9, 12, 16]. 

Subjective cognitive complaints, in addition to predicting the onset of a neurodegenerative process, may yield important information about areas of impairment, specifically in patients with mild disease. Two such disorders are mild cognitive impairment (MCI) and its vascular equivalent, vascular mild cognitive impairment (VaMCI). Differences in functional impairment between individuals with MCI and those with VaMCI, which may be overlooked by objective cognitive testing, may be predicted by an analysis of significance of SCC. In the current study, we compared the profile of SCC in individuals with a diagnosis of MCI to those with VaMCI using the Neuropsychological Impairment Scale (NIS). The NIS is a comprehensive scale-based questionnaire, which may elicit differences in presence and severity of SCC between the two groups. We hypothesized that the severity of subjective cognitive complaints would not differ between the two groups, because objective cognitive functioning is similar. However, we hypothesized that the specific types of SCC might differ between MCI and VaMCI, due to the differing pathologies of these two disorders.

## 2. Methods

### 2.1. Participants

Twenty patients with MCI and twenty patients with VaMCI matched on demographic characteristics (age, education) were recruited from the Memory Disorders Clinic at St. Michael's Hospital in Toronto. Patients are referred to the clinic by their primary or secondary care physicians, for assessment and diagnosis of cognitive impairment. Patients typically undergo a standardized history and a standardized cognitive exam as part of the workup. In addition, routine blood work including TSH, B12, and RBC folate is done to rule out reversible causes of dementia. As well, most patients receive structural imaging (either Computed Tomography (CT) or Magnetic Resonance Imaging (MRI)) and in some cases functional imaging (Single Photon Emission Computed Tomography (SPECT)). For this study, only patients clinically diagnosed with either MCI or VaMCI were recruited. This study was approved by the Research Ethics Board at St. Michael's Hospital.

The diagnostic criteria for MCI describe cognitive impairment which is more severe than normal aging but less severe than dementia and typically not associated with serious impairment in everyday functions [17]. VaMCI is described as the vascular equivalent of MCI, thereby indicating a similar level of objective cognitive performance despite possible differences in mechanism and etiology [18]. Currently, there are no specific neuroimaging or vascular criteria that are necessary for a diagnosis of VaMCI [18]. Our study population underwent clinical CT scans in most cases. Some patients underwent MRI scans instead of CT scans as part of their clinical visit. As we used different imaging modalities we could not compare degree of white matter disease across study subjects in a quantifiable manner. Rather, patients were assigned to the VaMCI group if scans showed evidence of white matter lesions, lacunar infarcts, and/or moderate to severe microangiopathic change [19]. However, patients with mild microangiopathic changes were not considered to have VaMCI. Cognitive testing and data intake occurred during the patients' initial visits to the clinic. Follow-up visits to the Memory Disorders Clinic were not considered for this cross-sectional analysis.

### 2.2. Primary Outcome Measures

#### 2.2.1. Subjective Measures

Subjective cognitive complaints were assessed using the Neuropsychological Impairment Scale (NIS) [20, 21]. The NIS is a questionnaire consisting of 95 complaints, such as “I am forgetful” and “I am easily distracted.” Subjects rated these statements on a five-point Likert scale according to applicability and intensity. When scored, the NIS divides complaints into seven domains: critical items (e.g., head injury, stroke, and dizziness), cognitive efficiency (e.g., confusion, mental slowness), and attention, memory, frustration tolerance, verbal learning, and academic skills (e.g., counting change, learning new tasks). The NIS also includes three scores that give a general, comprehensive picture of complaints: the Global Measure of Impairment (GMI) score is a simple sum of all domain subscores; the Total Items Circled (TIC) score indicates how many complaints are reported; and the Symptom Intensity Measure (SIM) score indicates the average intensity of complaints. The NIS also includes three validity checks: defensiveness, inconsistency, and affective disturbance. Thus, the NIS can detect if subjects are overly defensive or inconsistent; and the affective disturbance validity check controls for levels of depression and other emotional disturbances. Any of these states could affect the interpretation and validity of results.

### 2.3. Secondary Outcome Measures

#### 2.3.1. Objective Measures

All subjects were assessed via cognitive and functional measures. The Mini-Mental State Examination (MMSE) [22] was used to measure cognitive performance from a general perspective. Detailed cognitive testing was done via the Behavioural Neurology Assessment (BNA) [23]. The BNA consists of five subcategories: memory, attention, naming, visuospatial function, and executive function. Scores on the various subcategories can pinpoint specific areas of cognitive impairment. This test has been validated in dementia [23]. 

Functional ability was objectively assessed using the Older American Resources and Services (OARS) Activities of Daily Living Questionnaire [24]. This questionnaire assesses fourteen activities of daily living (ADLs): seven basic ADLs (eating, dressing and undressing, grooming, walking, getting in and out of bed, bathing, and going to the bathroom) and seven instrumental ADLs (using the telephone, traveling, shopping, preparing meals, doing housework, taking medications, and handling money). Subjects were asked about their own ability to perform the above ADLs; where possible, informants who knew the subjects well were asked to supplement and verify responses. 

### 2.4. Statistical Analysis

Mean scores for each group (MCI and VaMCI) were compared via an independent samples *t*-test. Significance was defined as *P* ≤ 0.050. Age, education, and other demographic factors were also compared between the two groups. Statistical analysis was performed using SPSS 16.0 for Windows (2007).

## 3. Results

The MCI and VaMCI groups were matched on demographic measures such as age, years of education, and Hollingshead Two-Factor Index of Social Position (Table 1). Functional ability, as measured by the OARS questionnaire, was also similar between the two groups (*P* = 0.919) (Table 2).

### 3.1. Objective Cognitive Function

Objective cognition function was measured by the MMSE and the BNA (Table 2). MMSE scores were similar between the two groups (*P* = 0.330), which implies similar levels of general cognitive function. Overall BNA scores were also similar between the MCI and VaMCI groups (*P* = 0.177). Scores from subcategories of the BNA were also compared between groups to look for specific areas of cognitive impairment (Table 2). Performance on all subcategories of the BNA (attention, memory, naming, visuospatial function, and executive function) was similar between MCI and VaMCI groups. Performance on the individual tasks which comprise the above five subcategories was also similar between groups, with the exception of the “explaining proverbs” task (*P* = 0.034). On this task, which falls into the subcategory of executive function, MCI subjects averaged a better score than VaMCI subjects (3.15 compared to 2.30).

### 3.2. Subjective Cognitive Complaints

Despite similar performance on objective functional and cognitive measures (OARS, MMSE, and BNA), the MCI and VaMCI groups achieved markedly different results when subjective cognitive complaints were assessed by the NIS. In general, VaMCI subjects had significantly higher scores on the NIS than MCI subjects (Table 3); higher scores indicate greater SCC. The NIS Global Measure of Impairment (GMI) score gives a broad, comprehensive view of complaints. The VaMCI group averaged a GMI score of 117.0, compared to just 79.3 for the MCI group (*P* = 0.050). A qualitative analysis of the Total Items Circled (TIC) scores shows that VaMCI subjects had a greater number of complaints than MCI subjects (52.8 compared to 44.2); however this difference was not statistically significant (*P* = 0.136). The Symptom Intensity Measure (SIM) scores reveal that VaMCI subjects' complaints were greater in severity than those of MCI subjects (2.0 compared to 1.7); however, again, this difference was not statistically significant (*P* = 0.065). Thus, we cannot attribute the difference in GMI scores to just one cause. Rather, it is likely that difference in GMI scores stems from differences in both complaint quantity and complaint severity.

The specific nature of subjective complaints was also analyzed. Quantitatively, the VaMCI group had higher scores in all seven domains of the NIS (Table 3). Four of these domains yielded differences in scores that were statistically significant: critical items (*P* = 0.017), cognitive efficiency (*P* = 0.043), memory (*P* = 0.046), and verbal learning (*P* = 0.027). The NIS also includes three validity checks to test for defensiveness, affective disturbance, and inconsistency. In our sample of subjects, there was no statistical difference in any of the above variables between the MCI and VaMCI groups (Table 3), confirming that the complaints were valid. Although VaMCI subjects had qualitatively higher scores on the three validity measures, their scores were not significantly different from those of the MCI group.

## 4. Discussion

MCI and VaMCI are clinical conditions that precede the development of neurodegenerative disorders including Alzheimer's disease and vascular dementia, respectively. Apart from differences on neuroimaging there has been very little study of how these two clinical conditions differ in terms of their subjective and objective clinical profiles. Taken altogether, our findings from the MMSE, BNA, OARS, and NIS show that despite similar levels of functional ability and objective cognitive function, VaMCI subjects have significantly more SCC than MCI subjects. Our findings support the existing view of MCI and VaMCI as disorders with similar presentations with regard to objective function, but ultimately different etiologies.

As previously discussed, MCI and VaMCI subjects were matched on age, education, and social status and achieved statistically similar scores on the MMSE and BNA. The one component of the BNA that differed between the two groups was the “explaining proverbs” task; however, this seeming disparity may have resulted from differences in cultural background. In our sample of patients from the diverse city of Toronto, 17 out of 40 subjects (10 MCI and 7 VaMCI) reported English as a second language. Unfamiliarity with English proverbs certainly confounded this result and may explain this difference observed on the BNA. Leaving aside this single anomaly, our findings show that MCI and VaMCI subjects had comparable levels of objective cognitive function. However, our findings from the NIS questionnaire show that VaMCI subjects had more subjective cognitive complaints than their MCI counterparts. This discrepancy suggests that VaMCI patients are exhibiting particular cognitive deficits, which are not identified by the BNA, or that VaMCI patients are more sensitive to and aware of mild deficits in these particular areas. The nature of VaMCI-specific deficits may be determined from the domains in which VaMCI patients report greater complaints: critical items, cognitive efficiency, memory, and verbal learning. The critical items domain references events in a patient's history that are evident predictors of cognitive difficulty: dizziness, concussion, head trauma, stroke, and so forth. VaMCI subjects reported greater complaints in this domain than MCI subjects; this finding may be explained by simply considering the diagnostic criteria of VaMCI compared to those of MCI. As mentioned above, history of clinical stroke can differentiate VaMCI from MCI [18]. A VaMCI patient is more likely than an MCI patient to have experienced stroke or stroke-like symptoms, so he/she is more likely to have complaints in the critical items domain.

VaMCI subjects' greater complaints in the memory, verbal learning, and cognitive efficiency domains may be explained by examining the pathology of VaMCI, which differs from that of MCI upon neuroimaging. A study by Vannorsdall et al. found that white matter hyperintensities in both periventricular and subcortical regions correlated with poorer working memory [25]. Interestingly, a study by Soriano-Raya et al. found that deep white matter hyperintensities correlated with decreased performance on verbal fluency tasks, while periventricular hyperintensities were not associated with any particular domain [26]. White matter lesions have also been correlated with slowed information processing [25, 27]. Recent research has demonstrated that this decrease in processing speed may be specific to white matter lesions in the anterior thalamic radiation [28]. 

Our analysis may be hampered by the limitations of our cognitive testing methods. In this study, the MMSE and BNA may have suffered from ceiling effects, which would have masked any differences in cognitive function between the MCI and VaMCI groups. However, the advantages of the MMSE and BNA are numerous and informed our decision to use these tests. They are relatively easy for clinicians to administer as part of a standard visit. In addition, more sensitive cognitive testing would not have been possible at the study facility, given the amount of time allotted for a clinical visit. The differences we have found in SCC between MCI and VaMCI groups may suggest a need for alternate cognitive testing methods, which, unlike the MMSE or BNA, would elicit different results between the two groups and would not suffer from ceiling effects.

Our study has demonstrated that VaMCI patients may be exhibiting particular cognitive deficits in memory, verbal learning, and cognitive efficiency, separate from those exhibited by MCI patients. These deficits may be explained by the greater white matter burden in VaMCI patients, as compared to age-matched MCI patients. Further research should be done to elaborate on the nature of VaMCI-specific deficits, particularly in the domains we have identified in this study. As well, VaMCI-specific deficits should be related to neuroimaging findings, in order to associate deficits with particular locations and severities of white matter disease. Finally, tools for more sensitive cognitive testing must be developed, so that we may identify VaMCI-specific cognitive deficits.

## Figures and Tables

**Table 1:** Demographic characteristics of the study sample.

	MCI	VaMCI	*P* value (two-tailed)
Age	66.95	66.15	0.810
Years of education*	15.00	14.45	0.671
Hollingshead Two-Factor Index of Social Position**	29.94	32.53	0.644

*Some subjects did not disclose information regarding their educational background. Overall, 17 out of 20 MCI patients and 20 out of 20 VaMCI patients disclosed their number of years of education.

**17 out of 20 MCI patients and 19 out of 20 VaMCI patients disclosed their occupational information. Lower Hollingshead scores refer to higher index of social position.

**Table 2:** Performance on measures of objective functional ability and objective cognitive function.

	MCI	VaMCI	*P* value (two-tailed)
OARS (/28)	27.53	27.56	0.919
MMSE (/30)	28.25	27.53	0.330
Overall BNA (/114)	91.50	87.95	0.177
Attention subscore (/6)	3.60	3.70	0.853
Counting backwards by 7 s (/2)	0.60	0.55	0.836
Counting backwards by 3 s (/2)	1.30	1.30	1.000
Months backwards (/2)	1.70	1.85	0.451
Memory subscore (/25)	23.10	22.95	0.819
Orientation (/7)	6.65	6.30	0.123
Immediate recall (/9)	9.00	9.00	1.000
Delayed recall (/9)	7.45	7.60	0.805
Naming subscore (/30)	24.20	23.00	0.331
Naming objects (/10)	9.90	9.80	0.389
Animals (/20)	14.30	13.20	0.370
Visuospatial subscore (clock-drawing) (/15)	13.95	14.05	0.843
Executive function subscore (/38)	25.50	24.20	0.384
Similarities (/10)	9.35	7.90	0.220
Proverbs (/4)	3.15	2.30	0.034
Multiple loops (/2)	1.90	1.90	1.000
Alternating sequence (/2)	1.85	1.95	0.411
*F* words (/20)	10.65	10.00	0.535

**Table 3:** Presence and severity of SCC, as assessed by self-report on the NIS.

	MCI	VaMCI	*P* value (two-tailed)
Global Measure of Impairment	79.275	116.975	0.050
Total Items Circled	44.200	52.800	0.136
Symptom Intensity Measure	1.6981	2.0481	0.065
Domains			
Critical items	5.150	11.350	0.017
Cognitive efficiency	10.225	16.075	0.043
Attention	13.500	18.100	0.173
Memory	12.050	15.875	0.046
Frustration tolerance	7.550	10.150	0.231
Verbal learning	5.800	10.575	0.027
Academic skills	11.550	14.650	0.282
Validity measures			
Defensiveness	8.800	10.450	0.126
Affective disturbance	10.650	12.775	0.496
Inconsistencies	5.250	5.450	0.837
